# Circuit-level convergence of electronics and photonics: basic concepts and recent advances

**DOI:** 10.1007/s12200-022-00013-8

**Published:** 2022-04-28

**Authors:** Min Tan, Yuhang Wang, Ken Xingze Wang, Yuan Yu, Xinliang Zhang

**Affiliations:** 1 School of Optical and Electronic Information, Huazhong University of Science and Technology, Wuhan, 430074 China; 2 Wuhan National Laboratory for Optoelectronics, Huazhong University of Science and Technology, Wuhan, 430074 China; 3 School of Physics, Huazhong University of Science and Technology, Wuhan, 430074 China

**Keywords:** Integrated photonics, Electronics-photonics convergence (EPC), Scalability, Stability, Feedback

## Abstract

Integrated photonics is widely regarded as an important post-Moore’s law research direction. However, it suffers from intrinsic limitations, such as lack of control and satisfactory photonic memory, that cannot be solved in the optical domain and must be combined with electronics for practical use. Inevitably, electronics and photonics will converge. The photonic fabrication and integration technology is gradually maturing and electronics-photonics convergence (EPC) is experiencing a transition from device integration to circuit design. We derive a conceptual framework consisting of regulator, oscillator, and memory for scalable integrated circuits based on the fundamental concepts of purposeful behavior in cybernetics, entropy in information theory, and symmetry breaking in physics. Leveraging this framework and emulating the successes experienced by electronic integrated circuits, we identify the key building blocks for the integrated circuits for EPC and review the recent advances.

**Graphical Abstract:**

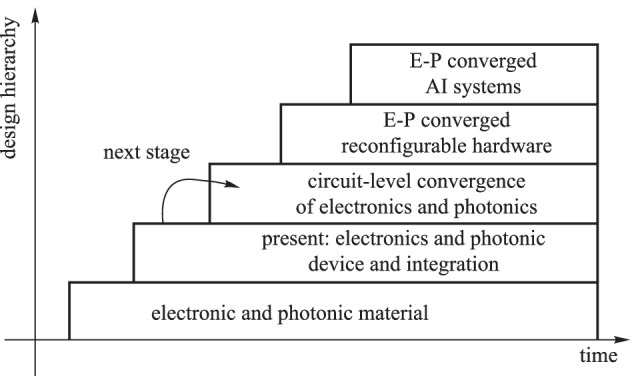

## Introduction

**Fig. 1:**
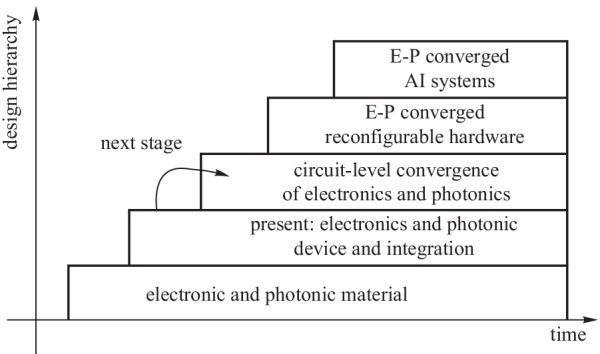
Design hierarchy of electronics-photonics (E-P) convergence

Moore’s law has been the main driving force for the information industry during the last half-century. However, due to physical and economic constraints, Moore’s law is approaching its end. Meantime, new applications (e.g., driverless cars, artificial intelligence (AI), and virtual reality (VR)) are emerging rapidly, which generates a large amount of data that results in capacity and energy efficiency challenges. Compared with electronics, photonics provides broad bandwidth and high energy efficiency. Photonic integrated circuits (PICs) are widely regarded as an effective solution to resolve these challenges [1]. This is exemplified in several applications, e.g., computer systems and data-center communications. Photonics has the potential to overcome the von Neumann bottleneck [2] of computer systems. Furthermore, neuromorphic photonics is considered a viable alternative to circumvent the von Neumann bottleneck [3]. Co-packaged optics (CPO) is considered indispensable for future high-speed switches [4]. In addition, a PIC provides new functionalities as it offers a new optical spectrum range and enables many new applications, e.g., solid-state LIDAR [5] and miniaturized spectrometer [6]. Nevertheless, a PIC alone without active control is essentially useless. It is widely believed that electronics and photonics will ultimately converge [7], and many institutions have established related projects. The AIM Photonics project [8], which is among the largest, aims to emulate the dramatic successes experienced by the electronics industry over the past 60 years and transition key lessons, processes, and approaches to the PIC industry. As shown in Fig. 1, electronics-photonics convergence (EPC) will go through a similar development hierarchy of microelectronics from materials to circuits to more complicated systems, such as AI systems. As the photonic fabrication and integration technology gradually matures, EPC is now transitioning from device integration to circuit design. Leveraging the development experience of the electronic integrated circuit (EIC) will accelerate the transition process of EPC.

Nevertheless, there are some key differences between EIC and the integrated circuit (IC) for EPC that prevents us from directly applying the knowledge of EIC design to EPC IC design. Electronic circuits deal with electronic signals only, while EPC circuits involve both electronic signals and photonic signals. In other words, electronic circuits deal with signals in the same domain, while EPC circuits deal with signals in two different domains. Note that a hybrid IC already has a reserved definition [9], and using the term heterogeneous IC helps avoid confusion with existing terminology. The techniques developed for conventional ICs may not be applied to heterogeneous ICs directly. For example, we can compare two electronic signals, but a direct comparison between electronic signals and photonic signals is either impossible or meaningless. Nevertheless, they share some key features at a more fundamental level. For example, all practical electronic circuits and EPC circuits are designed to perform a purposeful behavior, a concept that sets the bases of cybernetics [10, 11]. We derive a conceptual framework consisting of regulator, oscillator, and memory for scalable integrated circuits based on the fundamental concepts of purposeful behavior in cybernetics, entropy in information theory, and symmetry breaking in physics. This conceptual framework can be regarded as a metacircuits framework based on which concrete and complicated circuits can be generated. Leveraging this framework and emulating the successful experience of EIC design allows us to construct an evolution roadmap for the circuit-level convergence of electronics and photonics.

The remainder of this paper is organized as follows. Section 2 discusses the design challenges of integrated photonic devices and the necessity of circuit-level convergence of electronics and photonics. Section 3 presents the general conceptual framework for scalable ICs. Section 4 discusses the details of circuit-level convergence of electronics and photonics. Section 5 reviews the advances in the key building blocks of EPC ICs. Section 6 provides the future perspectives. Section 7 draws the conclusion.

## Design challenges of photonic devices

Due to both economic and physical limitations, it is impossible to make a perfect device. There always exist tradeoffs between the different performance metrics, and no perfect device exists. Synthesizing a reliable system out of unreliable elements is a common practice [12]. Electronic devices suffer from many intrinsic limitations (e.g., noise, process variations, temperature changes, and aging effect) that cannot be solved at the device level, and circuit-level techniques (e.g., analog circuit, radio frequency (RF) circuit, and digital circuit) have been invented to address these limitations. Autozero and chopper techniques were used [13, 14] to reduce offset and input noise. Miller compensation was used to reduce the capacitor size. Differential amplifiers were adopted to reduce common-mode noise. Photonic devices also face many design challenges. Some of them are intrinsic in the sense that they cannot be solved by improving the fabrication technology, e.g., wavelength variations of micro-rings. Progressing to a higher design hierarchy is the only way to overcome these intrinsic challenges. In this section, we discuss the key design challenges of photonic devices. A partial list of them includes on-chip laser, optical loss, device size, bandwidth, and stability. Stability is the central challenge and is usually traded off with other performance metrics.

### On-chip laser

Silicon is an indirect bandgap material, incapable of emitting light efficiently. Existing approaches for light integration include, III-V-to-silicon bonding [15], heterogeneous III-V-on-Si lasers [16], transfer printing [17], and epitaxial growth [18]. Most practical photonic systems use external light sources. Though there has been much research on light generation, little attention is paid to peripheral electronics. For a complete laser system, most of the space is taken by peripheral electronics. Reducing the area of the control electronics is required to enable high-density integration of laser arrays. Circuit-level convergence of photonics and electronics is essential to achieving this goal. Recent advances toward this goal include the integrated Pound–Drever–Hall laser stabilization [19].

### Propagation loss

It is always beneficial to reduce the propagation. Propagation loss in silicon waveguides is mainly due to light scattering on the sidewall roughness of the waveguide [20]. For strip waveguide, the propagation loss can be made < 2 dB/cm without too much effort. Achieving < 1 dB/cm loss is challenging but possible with special techniques such as H_2_ thermal annealing [21]. Compared with silicon photonics, silicon nitride achieves a much lower loss and also extends the usable spectral window toward 405 nm [22].

### Device size

There is no Moore’s law for integrated photonic devices as the device size of photonic devices needs to match the wavelength. Also, to achieve sufficient electro-optic interaction, the device cannot be made too small. For example, sufficient arm length needs to be maintained to reduce the driver voltage. A number of techniques have been proposed to reduce device size, e.g., subwavelength devices using inverse design [23] and plasmonic devices [24]. However, these techniques result in performance degradation in other aspects, such as high loss, reduced robustness, and limited application scope, and currently are not used in practical applications.

### Bandwidth

Optical spectrum covers a wide frequency range, but optical devices have limited bandwidth, especially active devices, such as modulators and detectors. Also, in many cases, the operating bandwidth is a combined effect of photonics and electronics. For example, the performance of the transmitter depends on both the modulator and the driver IC. The receiver performance depends on both the photodiode and the trans-impedance amplifier. In a Mach–Zehnder modulator (MZM), the EO bandwidth critically depends on the RF loss of the electrode [25]. Therefore, a converged design of electronics and photonics is required to optimize the bandwidth.

### Stability

Due to fabrication errors and environmental changes, photonic signal intrinsically suffers from phase uncertainty. All phase-sensitive devices (e.g., micro-rings and MZIs) suffer from stability problems, and therefore, are imperfect devices. Almost all practical applications contain one or more phase-sensitive devices and suffer from photonic parameter stability. Taking micro-ring as an example, its wavelength dependence on waveguide thickness and width is roughly 1 nm/1 nm [26]. The thickness and width variations can easily go beyond 3 nm. Silicon has a lattice parameter of 0.543 nm. Even with atomic fabrication precision, the wavelength granularity will be 0.543 nm, not fine enough to support practical applications that require a higher wavelength accuracy. Actually, most applications require a fine wavelength accuracy, e.g., 10 pm wavelength accuracy. It is theoretically impossible to solve the wavelength accuracy problem by improving the fabrication technology alone. Active tuning is the only way to achieve the desired accuracy.

**Fig. 2:**
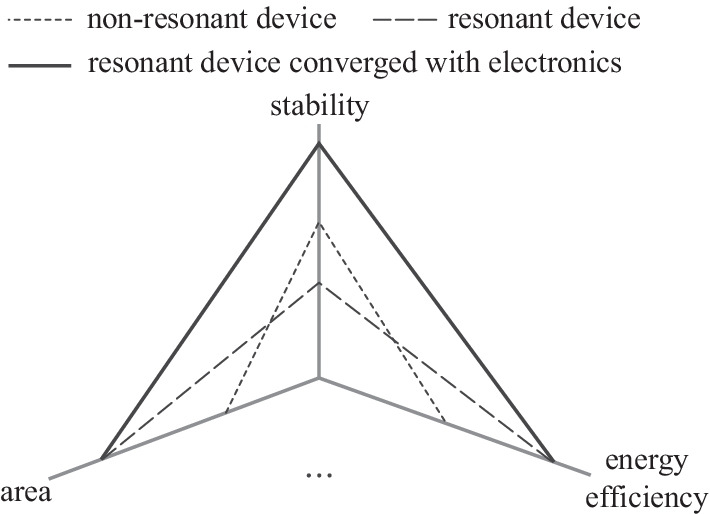
Trade-offs between stability and other metrics for resonant devices

The above challenges are closely related to each other and there exist tradeoffs between them. Improved stability can relax the requirements on other performance metrics. We use cavity-enhanced devices [27], more specifically micro-rings, to illustrate this point. As shown in Fig. 2, resonant devices usually achieve better performance at the cost of stability. Once the stability problem is solved by converging it with electronics, the resulting circuit is likely to outperform its non-resonant counterpart in all performance metrics. The cavity is a light-matter interaction enhancement method with a wide range of applications, but its resonant wavelength suffers from the stability problem. Micro-ring is a versatile cavity device that finds applications in laser, modulator, photodetector, photonic signal processing, etc. Compared with MZI modulators, micro-ring modulators are more energy- and area-efficient. Resonant micro-ring photodetectors achieve better responsivity [28]. Micro-ring is the key component in external cavity semiconductor lasers [29] and will play an important role in emerging integrated photonic neural networks [29]. Enhanced performance is achieved at the price of wavelength stability. If stability is maintained, the cavity-enhancing approach usually outperforms its non-resonant counterpart. The electronic-photonic hybrid feedback is the only practical way to resolve the cavity stability problem. We discuss this point in more detail in Sect. 4.

## General conceptual framework for scalable integrated circuits

**Fig. 3:**
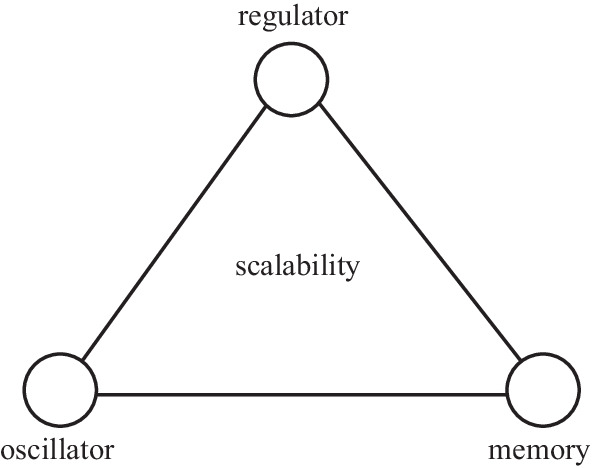
Scalability triangle for general scalable integrated circuits (ICs)

Integrated circuits (ICs) are designed to execute human intentions. In other words, they perform purposeful behavior. Purposeful behavior is a fundamental concept that sets the bases of cybernetics [30]. Feedback behavior forms an indispensable part of purposeful behavior [30] and is required for most practical applications. A regulator is used to execute the stabilization operation. High scalability is required when there is a large amount of data, and is one of the key challenges of the information industry. Data contains information if it is considered meaningful. Big data contains a large amount of information. From information theory, information is about the resolution of uncertainty which is measured by entropy [31]. Static signals have no uncertainty, hence no information. Dynamic signals can either be periodic or aperiodic. There are infinitely many aperiodic signals, and it is almost impossible to build separate circuits for the generation, processing, transmission, and detection of each aperiodic signal. Using periodic signals is a better choice. However, pure periodic signals with symmetry patterns contain no uncertainty, hence no information. Information originates in symmetry breaking [32–34]. To create information, one needs to break the symmetry by modifying the periodic signals using other signals, which can either be external input signals or readout signals from internal memory. As we want to minimize the external inputs, memory is usually required for large-scale systems. Memory allows us to construct sequential logic. Feedback is the prerequisite for stability, oscillation, and sequential logic, and a feedback-centric design framework becomes a natural choice for any integrated circuit that performs purposeful behavior. As shown in Fig. 3, regulator, oscillator, and memory form a scalability triangle that underpins a conceptual framework for scalable ICs. Essentially this conceptual framework can be regarded as a metacircuits framework based on which concrete and complicated circuits can be generated. Not all integrated circuits can be generated from this metacircuits framework. However, more complex ICs usually consist of these three key elements.

We show in the next section that the scalability triangle exists in the electronic domain. This scalability triangle forms the core of analog circuits, RF circuits, and digital circuits. EIC is a scalable IC, and more complicated systems can be generated from these three types of circuits. The electronic industry eco-system is based on these three types of circuits. Devices in other signal domains usually fail to construct the complete scalability triangle and must be merged with electronic devices to achieve scalability. For example, photonic devices do not have satisfactory photonic memory, and therefore, will have limited scalability.

## Circuit-level convergence of electronics and photonics

Emulating the successful experience of the microelectronics industry benefits the development of EPC across all levels of the design hierarchy. At the material level, the phase-changing property of materials is first explored in electronics [35] and then studied in photonics [36]. At the device theory level, photonic bandgap [37] is inspired by electronic bandgap. At the device fabrication and integration level, the fabrication of photonic devices has benefited greatly from the fabrication infrastructure of microelectronics. Similarly, electronic circuit design serves as a good reference for EPC circuit design. However, it remains to be answered how we transition the key lessons, processes, and approaches of microelectronics to EPC at the circuit level. Before answering this question, let us first examine the concepts of PIC [38–40], optoelectronic integrated circuit (OEIC) [41, 42], electronic-photonic integrated circuit (EPIC) [43] and discuss their relationship with the circuit-level convergence of electronics and photonics. Existing research on PIC, OEIC, and EPIC mainly focused on device fabrication and integration and did not study the scalability triangle of the regulator, oscillator, and memory. For this reason, none of them can be regarded as the scalable circuit technology for EPC to achieve purposeful behavior. The EPC IC design technology is yet to be developed, and new circuit concepts are required for the EPC [7]. We name this new type of IC for EPC electronic-photonic heterogeneous-converging IC (EPHIC). There are several reasons for coining this name. First, using a new name helps us distinguish it from PIC and EPIC, which mainly focus on device fabrication and integration. Furthermore, monolithic electronic-photonic integrated circuits may not be available for mass production for many years to come due to both economic and technological reasons. CMOS compatible laser integration will be very challenging, if not impossible. Finally, to emphasize that the converging process is a long-term evolution, we name the IC for EPC as electronic-photonic heterogeneously-converging IC (EPHIC). EPHIC is built upon EIC and PIC but focuses more on the interaction between electronics and photonics. In the remainder of this section, we first review the experience of EIC design and discuss the key elements of EPHIC.

### Successful experience of the electronic integrated circuits design

The scalability triangle consisting of regulator, oscillator, and memory is a general conceptual framework for building scalable ICs. As EIC is a scalable technology, it must have the three elements of the scalability triangle. In EIC, stability is achieved by a regulator. In this case, a feedback loop is established, and the output voltage is regulated to be the expected value. A regulator can be regarded as an amplifier connected in unity-gain feedback configuration, which can take various forms such as linear amplifiers, switching amplifiers, and hybrid amplifiers. The amplifier is widely regarded as the most fundamental building block for constructing more complex functions. An oscillator generates periodic signals and is the key building block for radio-frequency integrated circuits (RFICs). The first integrated circuit is the oscillator built by the Nobel prize winner Jack Kilby. For an oscillator to work, a feedback loop is required. For linear systems, the Barkhausen criterion is the necessary condition for oscillation generation [44]. A second feedback loop is usually required to stabilize the oscillation frequency. In sequential logic, the previous output signals are stored in memory and then fed back to the inputs to participate in the current operation. Memory is essential to this delayed feedback operation. In practice, combination logic is rarely used alone and is usually combined with sequential logic to form large-scale systems.

**Fig. 4:**
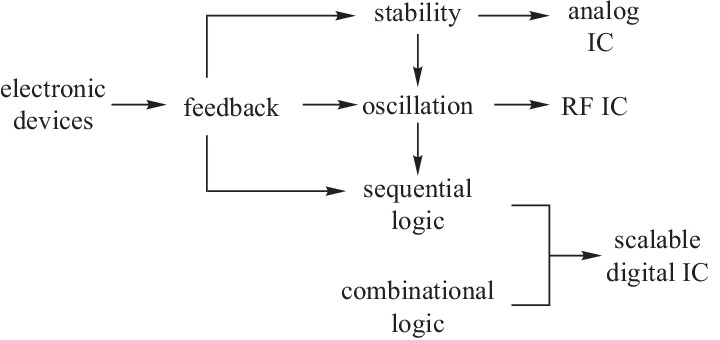
Evolution roadmap for the transition from devices to electronic integrated circuit (EIC)

Figure 4 shows the EIC evolution roadmap from devices to circuits. There are different types of electronic devices including passive device, active device, memory and so on. Feedback and memory form the foundation for stability, oscillation, and sequential logic, which in turn form three basic types of ICs, namely analog IC, RF IC, and digital IC. Not all circuits can be classified into the three basic types. However, more complex ICs usually consist of these three types of circuits. With Moore's law, we have transistors with smaller size, lower cost, and better performance. Modeling and electronic design automation (EDA) tools greatly speed up the design process and increase the level of complexity that we can handle.

### Key elements of EPHIC

EIC has the complete scalability triangle, but PIC alone only has the oscillator, that is, the laser. There is no practical photonic memory that plays a similar role of the register in EIC as stationary photons do not exist. Furthermore, there will be no practical all-optical feedback parameter regulation. EPHIC heterogeneous regulators stabilize the photonic signal using a hybrid feedback loop. Furthermore, a hybrid feedback loop can generate periodic signals, which can either be electronic signals or photonic signals.

**Fig. 5:**
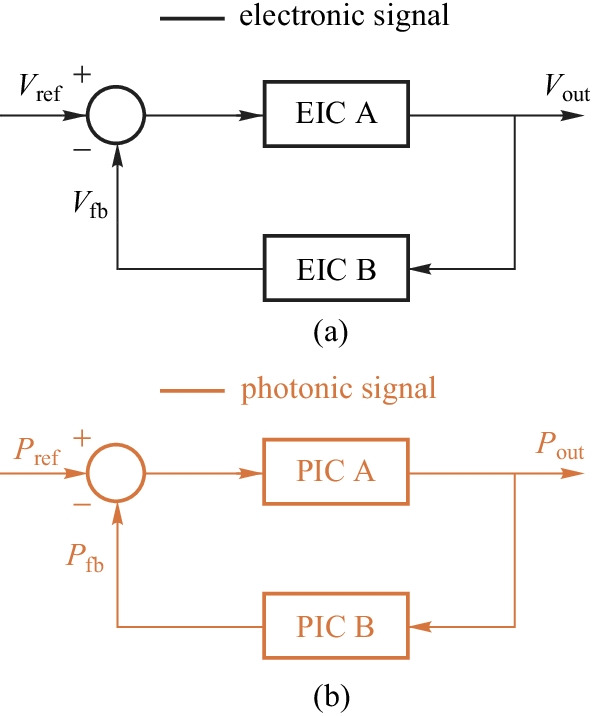
**a** Feedback stabilization of electronic signal. **b** All-optical feedback stabilization of photonic signals

**Fig. 6:**
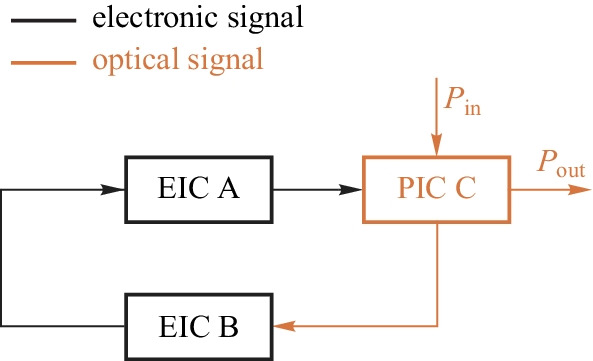
Photonic parameter stabilization via hybrid feedback

Figure 5a shows the block diagram of the feedback stabilization of electronic signals. Analog EIC has developed a solid foundation for the stability of regulators, from theory to implementation and to design automation tools. Nyquist theorem forms the foundation of stability. Various control architectures have been developed in the literature, including linear, switching, hybrid architectures and so on. Is it possible to replicate the design of EIC stability for the design of photonic signals, as shown in Fig. 5b? For this approach to succeed, first of all, a stable all-optical reference signal is required. We then need to have all-optical comparators, all-optical signal processors, and all-optical tuning methods. This approach is not like to work, if not impossible at all, before we have stable and practical on-chip lasers. Electronic-photonic (EP) hybrid feedback regulation is so far the only practical approach for photonic parameter stabilization. Figure 6 shows the conceptual block diagram for EP hybrid feedback. Instead of using an absolute reference for signal stabilization, EPHIC uses a self-referencing approach for photonic signal stabilization as photonic signals cannot be compared directly. In some sense, EIC uses an absolute reference framework, while EPHIC uses a relative reference framework. This is the fundamental difference between EIC and EPHIC. The main idea of photonic signal stabilization is to monitor the status of photonic devices, and regulate it to the expected status via EP hybrid feedback control. The situation is much more complicated than the EIC case. One may wonder how the existing photonic systems solve the stability problem. Existing systems indeed have solved this problem to some extent. However, for integration to be meaningful, we need to solve this problem with limited on-chip resources, which creates many new challenges. Utilizing the wavelength locking of a micro-ring as an example, we cannot afford high-performance ADCs, DACs, and expensive FPGAs/DSPs in the controller. Currently, there are no mature solutions for these new challenges.

RF EIC has developed a solid foundation for electronic oscillators. PIC also has its oscillator, that is, the laser. However, the laser resonant frequency drifts due to fabrication variations and environmental changes. EP hybrid frequency stabilization is required to fix the resonant frequency. It is a heterogenous frequency regulator as both electronic and photonic signals exist in the same loop. There are other approaches to generate or stabilize periodic signals via the electronic-photonic feedback loop, e.g., opto-electronic oscillator (OEO) [45], optical phase-locked loop [46], electro-optic phase-locked loop [47], and optical frequency stabilization [19]. More details are provided in the next section.

**Fig. 7:**
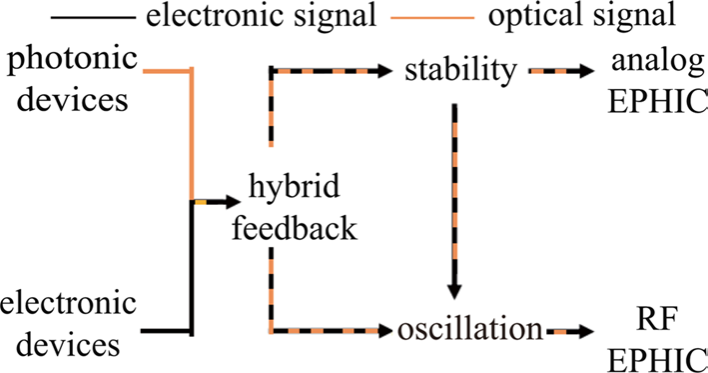
Evolution roadmap for the transition from devices to EPHIC

Figure 7 shows the evolution roadmap for the transition from devices to EPHIC. EPHIC includes PIC and EIC as the foundation but focuses more on the interaction between electronics and photonics. Both signal stabilization and oscillation exist in EPHIC. Hybrid feedback is required for heterogeneous regulators and heterogeneous oscillators, which form the core of analog EPHIC and RF EPHIC, respectively. Heterogeneous regulators and heterogeneous oscillators have both electronic signals and photonic signals in a closed loop.

Unlike electronic digital circuits, the signal strength of optical digital circuits attenuates along the propagation path and cannot be restored to its original value. In some sense, the optical digital circuits are essentially analog photonic circuits. There exists no practical photonic memory that resembles the role of register in EIC. Without practical photonic memory, there will be no practical sequential optical digital circuits. Furthermore, there is no scaling law for integrated photonic devices that play the similar role of Moore’s law in microelectronics as the device size needs to match the wavelength. Compact modeling methods and electronic-photonic design automation (EPDA) tools, are urgently needed to accelerate the converging process of electronics and photonics.

## Recent advances in the key building blocks of EPHIC

The interaction between electronics and photonics creates new heterogeneous building blocks, which can be classified into two types according to their functions: (1) photonic parameter stabilization and (2) oscillation generation and stabilization. Currently, most existing designs use PCB solutions. However, PCB solutions have limited scalability, and moving into chip solutions for EPC is inevitable. We will briefly review the recent advances in these two types of designs.

### Photonic parameter stabilization

**Fig. 8:**
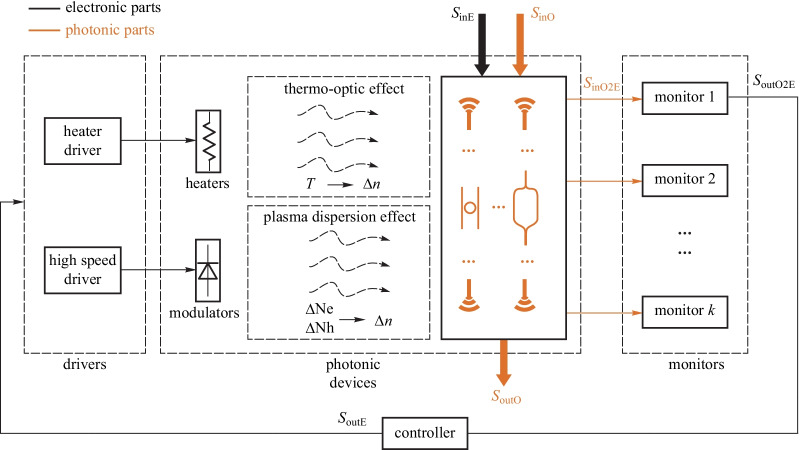
General model of the photonic parameter regulator. Adapted from Ref. [48]

The heterogeneous regulator for photonic parameter stabilization aims at keeping the photonic parameters away from fabrication errors and environmental changes using feedback control. The hybrid feedback is essential to the stabilization of optical parameters. Figure 8 shows the general model of the heterogeneous regulator. Many parameters of photonic devices need to be stabilized, e.g., resonance wavelength of micro-ring (MR) filters and the bias point of Mach–Zehnder modulators (MZMs). This regulator may have multiple open-loop electronic input signals and multiple closed-loop electronic input signals. For example, a micro-ring modulator has a high-speed open-loop driving signal and a closed-loop power input signal for the thermal phase shifter.

The monitors acquire the status information of the photonic devices, e.g., resonance wavelength of a micro-ring resonator and bias point of the MZM. Commonly used monitors include: (1) on-chip photodetectors (PDs) to monitor the light intensity at the output ports [49–54]; (2) on-chip temperature sensor [55–57]; (3) in-resonator photoconductive heaters (IRPHs) to monitor the light intensity in the waveguide [58–60]; (4) contactless integrated photonic probe (CLIPP) to measure the light-intensity-dependent change of the electronic conductivity of the waveguide [61–63]. The monitoring information is further processed by the controller using some basic algorithms, e.g., dithering [64], locking to maximum or minimum [49, 53], locking to reference [65–68], and so on. Then, the processed signal is passed to the drivers for thermo-optical tuning based on thermo-optical effect or electro-optic tuning based on plasma dispersion effect, finishing the closed-loop regulation cycle.

#### Wavelength regulator for micro-ring filters

**Fig. 9:**
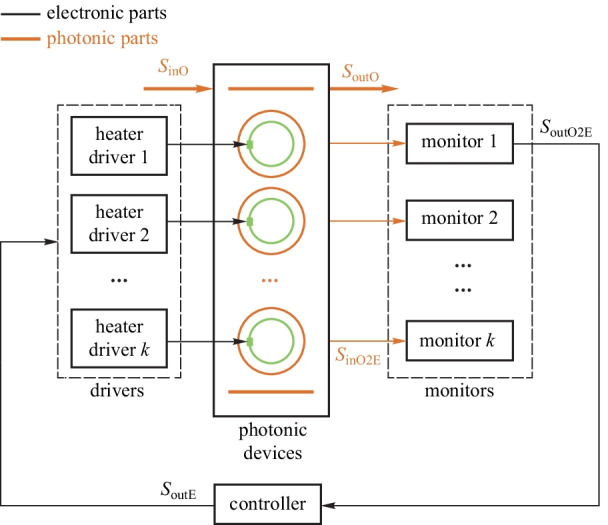
General model of wavelength regulator for micro-ring (MR) filters. Adapted from Ref. [48]

MR filters attract wide attention due to their compact size and low power consumption. However, their resonant wavelength will drift due to thermal fluctuations, input laser fluctuations, and process variations. Stable operations of MR filters can only be achieved with electronic-photonic hybrid feedback control. The general block diagram is shown in Fig. 9. The resonant wavelength is acquired by the monitors and is then adjusted to the expected wavelength by the heaters inside the MR. The wavelength regulator maintains the stable filtering operations of the MR filters.

In Ref. [69], the resonant wavelength of a high-order MR filter is locked by a board-level controller. The locking procedure is achieved by adjusting the heater of each MR one by one to maximize the drop port’s optical power. The potential issue of this scheme is the long iteration time and convergence problem. In Refs. [58, 59], the automatic configuration and wavelength locking of each MR is achieved based on IRPHs, which can simultaneously monitor and tune the resonance of each MR. However, the thermal crosstalk between adjacent MRs will increase the iteration time of the controller. In Ref. [70], a thermal eigenmode decomposition (TED) method is adopted, which can reduce the number of iterations of the controller. However, the demand of simultaneously tuning of each heater in different MRs makes the controller too complicated to be applied to large-scale systems. In Ref. [71], a fully integrated pulse width modulation (PWM)-driven closed-loop wavelength locking circuit for MR is first demonstrated with better power efficiency than a linear driver, but the stability, accuracy, and speed of the closed-loop PWM control scheme remain to be further improved.

The main challenges of the wavelength regulator for MR filters are efficient tuning of optical devices and slow iteration speed or convergence problem of the controller. In the future, the wavelength regulator for MR filter can be applied to microwave photonic filters with high *Q* factor and high-performance MUXs/DEMUXs.

#### Wavelength regulator for Si micro-ring modulators

**Fig. 10:**
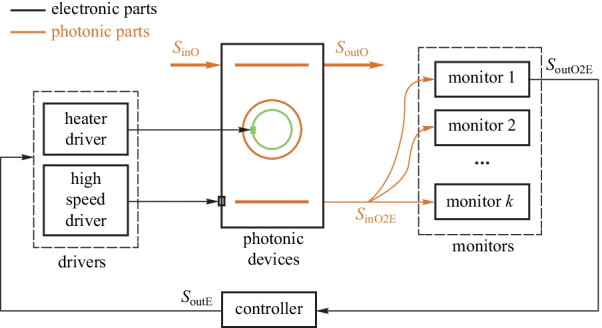
General model of wavelength regulator for Si MR modulators. Adapted from Ref. [48]

The silicon MR modulators achieve high modulation bandwidth with compact size and low power consumption. However, the resonant wavelength of the MR modulator is sensitive to thermal fluctuations, input laser fluctuations, and process variations. Stable modulation is only possible if wavelength stability is maintained. A wavelength regulator is essential to proper operations of MR modulators. The general model is shown in Fig. 10. The monitors acquire the current status of the MR modulator related to the optical signal from the MR modulator and pass the status information to the controller. The controller calculates the output value through an appropriate locking algorithm and tunes the resonant wavelength to the optimal point using heaters to achieve wavelength regulation.

In Refs [66, 72], the temperature change of the micro-ring modulator is detected using the mean power of the modulated output signal. However, this method is not suitable for non-DC balanced data sequence and unstable input optical power conditions. In Ref. [52], the resonant wavelength of the micro-ring modulator is locked by a maximum optical modulation amplitude (OMA) locking algorithm. However, the demand for manual delay adjustment and a specific data transmission sequence limits its application. In Ref. [73], the bit-statistics method achieves wavelength locking of an MR modulator under random data sequences. However, a specific training data sequence is still needed. Finally, in Ref. [74], an average-power-based thermal wavelength control circuit is proposed, which is immune to input laser power fluctuations. However, this method is still not suitable for non-DC-balanced NRZ data [75].

The main challenges of the wavelength regulator for MR modulator are (1) monitors that can deal with fast laser power fluctuations; (2) controllers that can achieve fast and stable control; and (3) heater drivers that can achieve precise and low power heater tuning. In the future, the wavelength regulator for MR modulators will achieve error-free operation and pave the way to high-performance optical connections.

#### Bias regulator for Mach–Zehnder modulator

**Fig. 11:**
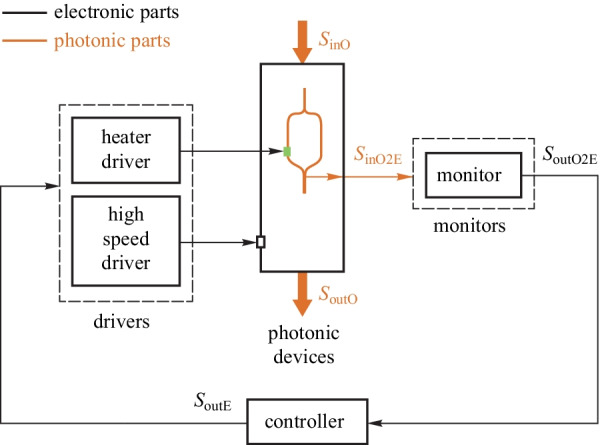
General model of bias regulator for Mach–Zehnder modulator. Adapted from Ref. [48]

Mach–Zehnder modulator is a widely used high-speed electro-optic modulator. However, its bias point will drift over time due to various factors (e.g., temperature changes). This drift will degrade the stability and quality of the modulated signal. By adjusting the phase shifters on the arms of the MZM through closed-loop bias control, the bias drift can be canceled out. The general model is shown in Fig. 11. The monitor extracts the current bias point. The controller adjusts the heater voltage according to the current bias point. A stable bias regulator system is then achieved.

In Refs. [76, 77], the output optical power or the derivative of output optical power is used to determine the current bias point. However, this method is sensitive to the input laser power fluctuation. In Ref. [78], the dithering (pilot tone) method is adopted to eliminate the influence of the input optical power fluctuations. By monitoring the harmonic components generated by the dithering signal, the status of the current bias point is extracted. This method will introduce external disturbance (the dither signal) to the modulator and may degrade the output signal quality. In Ref. [79], the OMA method is adopted to determine the current bias point. However, the OMA monitor consumes too much power. Besides, the OMA monitor method can be combined with the output power monitor method to reduce power consumption [80]. Using the OMA monitor method with a max search control algorithm to find the optimal bias point and lock it by output power monitor method with PID control algorithm, power-efficient bias control schemes can be achieved. In Ref. [81], the dither-correlation detection method is proposed to increase the monitoring sensitivity. However, this method is too complicated for on-chip integration. In Ref. [82], the power dither method and cross-correlation integral operation is proposed to compensate for the nonlinearity of the thermal phase shifter.

Up to now, most bias control schemes are achieved by using discrete devices. This approach has large power consumption and occupies a large area. Only a few works are targeted at silicon MZMs. An integrated bias controller for silicon MZMs will be the key to large-scale electronic-photonic convergence and is under active investigation.

#### Polarization regulator for fiber-to-PIC coupling

**Fig. 12:**
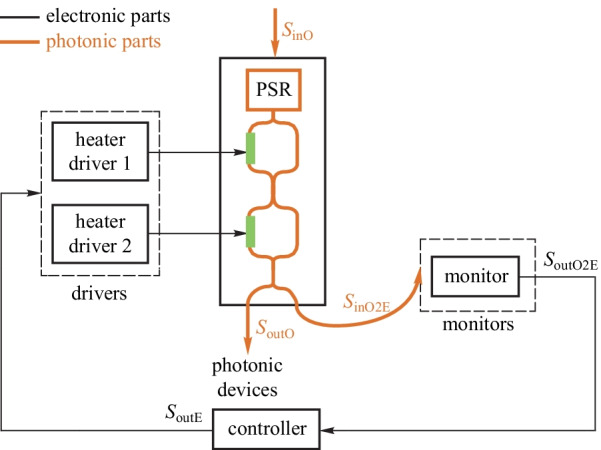
General model of polarization regulator for fiber-to-PIC coupling. PSR: polarization splitter-rotator. Adapted from Ref. [48]

The polarization of light in the fiber is sensitive to various factors such as external disturbance and internal asymmetry, resulting in an unstable and time-varying polarization state at the fiber output. The polarization mismatch between the SMF and the PIC degrades the signal quality. Active polarization control is a promising solution for the polarization mismatch problem. By compensating the magnitude and phase mismatch between two polarized components and combining them into one output, arbitrary input polarization state can be converted into a fixed on-chip polarization state. The hybrid feedback control is required for real-time tuning and time-varying compensation. The general model is shown in Fig. 12. The monitor extracts the output power of the feedback port. The controller adjusts the heater voltage according to the current output power in the feedback port to maximize the output power in the output port. The real-time polarization regulator for fiber-to-PIC coupling is then achieved.

In Ref. [83], a proof-of-concept prototype is demonstrated, but manual control is used to verify the concept. In Ref. [84], an active polarization control scheme is adopted in the WDM link. In Ref. [85], a new control algorithm in the active polarization control is proposed, verified, and compared with the previous control algorithm. In Ref. [86], active polarization control is combined with wavelength control of micro-ring filters to realize a tunable WDM polarization-independent receiver. In Ref. [87], an endlessly adaptive polarization controller based on a Mach–Zehnder interferometer is proposed, which can be used in coherent systems.

The main challenge of the polarization regulator is the endless and reset-free control. Both optical structure and the control algorithm need to be modified to eliminate the reset operation. The endless active polarization control scheme is likely to solve the polarization mismatch problem and eliminate the need for the off-chip bulky polarization controller.

#### Mode regulator for undoing strong mixing between modes

**Fig. 13:**
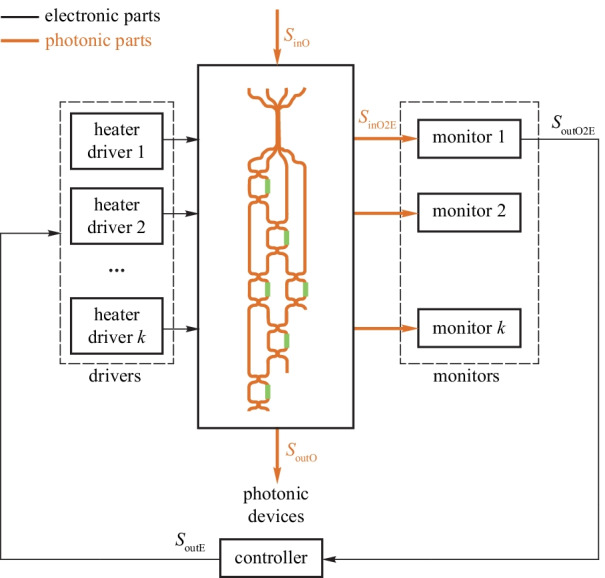
General model of mode regulator for undoing strong mixing between modes

The spatial coherence of the light will randomize due to propagation through multimode system [88]. The different mode lights will be mixed with each other and introduce the signal crosstalk. The mode crosstalk can be demultiplexed with an optical MIMO structure, consisting of Mach–Zehnder interference mesh. By building the proper optical transfer matrix, the strong mixing between modes can be undone. The proper optical transfer matrix can only be established with the help of electronic feedback tuning and locking. The optical MIMO structure and the electronic feedback tuning and locking circuits form the mode regulator. The general model is shown in Fig. 13. The monitor detects the current crosstalk between modes. The controller adjusts the heater voltage to modify the optical transfer matrix. The real-time mode regulator for undoing strong mixing between modes is then achieved.

In Ref. [89], a mesh of Mach–Zehnder interferometers (MZIs) realizes arbitrary unitary transformations and acts as an optical MIMO equalizer, and a self-configuration scheme is proposed. However, the configuration time is too long for practical use. In Ref. [88], transparent light detectors integrated with a photonic chip are used to monitor the evolution of each mode passing the mesh network, allowing sequential tuning and adaptive individual feedback control of each beam splitter. In Ref. [90], the modified self-configuration is proposed to speed the configuration time, but only simulation results are demonstrated.

The current mode regulator for undoing strong mixing between modes is still not good enough for practical use. The main challenges are high-performance optical devices and fast, stable configuration methods. The mode regulator for undoing strong mixing between modes can also be exploited to improve the performance of recently proposed silicon photonics devices for the manipulation of MDM optical channels.

### Oscillation generation and stabilization

In electronic-photonic hybrid system, the stable oscillation signals are always needed such as laser output with stable wavelength, the RF signal with ultra-low phase noise. These high-quality signals can be generated by the open-loop method by a high- performance device which is bulky and high-cost. Electronic-photonic hybrid feedback loop is an alternative method to generate these signals with low cost and compact size. By locking the output signal to a stable reference, stable oscillation signal can be acquired. Currently, the electronic-photonic feedback loop systems aimed at generating stable oscillation signal can be concluded as follows: opto-electronic oscillator (OEO), electro-optic PLL (EO-PLL), optical PLL (OPLL), and laser frequency stabilization (LFS). The main distinguishing feature and comparison are shown in Table 1.

**Table 1:** Electronic-photonic hybrid oscillation generation methods

	Output signal	Locked parameters	Reference
OEO	RF signal	Frequency of the RF signal	Optical delay
EO-PLL	Optical signal	Frequency modulated optical signal	Electrical reference
OPLL	Optical signal	Phase and wavelength of the slave laser	Master laser
LFS	Optical signal	Wavelength of the laser	Reference frequency

#### Opto-electronic oscillator

**Fig. 14:**
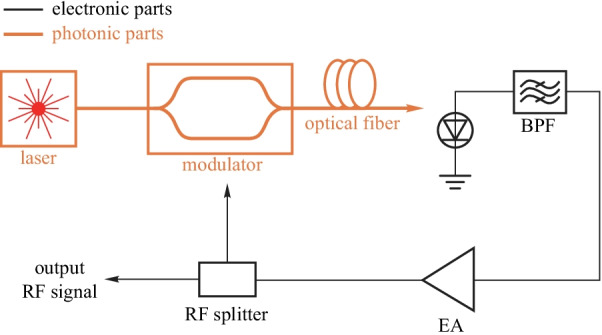
A simplified diagram of an OEO based on optical fiber

Opto-electronic oscillator can generate high-frequency RF signal with low phase noise. The frequency of the RF signal is determined by time delay in OEO which can be achieved by a long optical fiber or a high *Q* resonator [91]. The simplified diagram of a fiber-based OEO is shown in Fig. 14. A continuous-wave light emitted from a laser is modulated by an optical intensity modulator with RF signal. Then, the modulated signal passes the long fiber and acquires the time delay. The optical signal is further converted into electronic signal by the photodiode.

The desired signal is selected by the bandpass filter and amplified by an electronic amplifier (EA). The amplified signal is divided into two parts by the RF splitter. One part forms the output of the OEO, and the other part is sent to modulate the laser and close the loop.

**Fig. 15:**
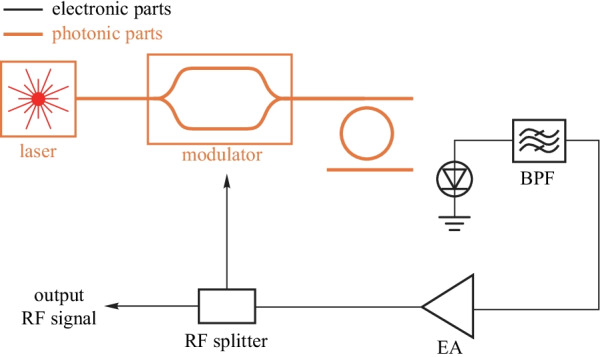
A simplified diagram of an OEO based on a high *Q* resonator

The time delay can also be realized by a high *Q* resonator to reduce the size and weight of OEO, which is shown in Fig. 15. The high *Q* factor is essential for low phase noise RF signal generation. The regulator modules form the fundamental part of the oscillator. When a MZM is used in OEO, the bias regulator of MZM must be adopted to achieve linear modulation [78]. Besides, a temperature stabilizer should also be used to stabilize OEO temperature. A piezoelectric transducer (PZT) based feedback control loop is needed to stabilize the loop length of OEO further. In a high *Q* resonator based OEO, the wavelength regulator for the resonator is essential for a stable oscillator.

The concept of OEO is first proposed by Yao and Maleki in 1996 [92]. The single loop structure is used to generate the single-frequency microwave signal. And various OEO structures are proposed since then such as multiloop OEO [93], resonators-based OEO [94], wideband frequency tunable OEO [95], etc. These schemes are based on discrete devices which are bulky and power hungry. To reduce the power consumption and the size, integrated OEO schemes are also demonstrated in silicon [96] and InP [97] platform which are more suitable for practical use.

However, the stability issue is still one of the main challenges for the practical demonstration of OEO. For stable RF signal generation, the regulators for optical delay lines, resonators, modulators and so on are still needed.

#### Electro-optic PLL

The frequency modulated continuous wave (FMCW) LIDAR requires less timing precision compared with pulsed LIDAR. However, the tuning nonlinearity and the temperature sensitivity degrade its performance [47]. The performance can be guaranteed by adopting the electro-optic PLL. The electro-optic PLL is based on electronic-photonic hybrid feedback and can achieve high-performance laser at a low cost.

**Fig. 16:**
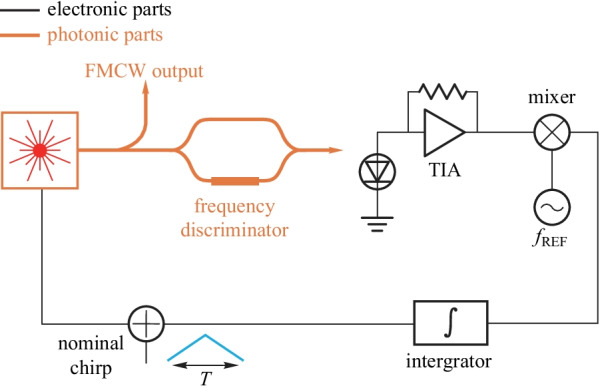
A simplified diagram of continuous-time electro-optic PLL

The simplified diagram of the continuous-time electro-optic PLL (CT-EOPLL) is shown in Fig. 16. The Mach–Zehnder interference (MZI) generates the beat signal, which is the product of the chirp slope and the MZI delay. The beat signal is mixed with the electronic reference frequency and then integrated by the integrator, whose output is added with the nominal chirp and then sent to the laser. Thus, the frequency of the beat signal can be locked to the reference frequency, and a stable oscillator is achieved. The bias regulator for MZI is also essential for stable oscillation.

The concept of the EO-PLL is first proposed in Ref. [98], where the rate and shape of the optical frequency sweep is locked to and determined by the frequency of a reference electronic signal. The EO-PLL can be also achieved by electronic-photonic integrated circuits which can reduce the size and cost [99]. Recently, highly integrated CT-EOPLL schemes are proposed which can achieve high-performance FMCW LIDAR [100, 101].

A fully integrated EO-PLL needs to solve the stability issue of the frequency discriminator which is formed by the MZI. The fabrication error and thermal fluctuation will greatly affect the accuracy of the MZI. A stable regulator for MZI is needed.

#### OPLL

Optical phase-locked loop is widely used in various applications, e.g., optical communication and microwave photonics. The OPLL can synchronize the wavelength and the phase between the master laser and the slave laser which makes it able to generate high-quality optical signal at a low cost.

**Fig. 17:**
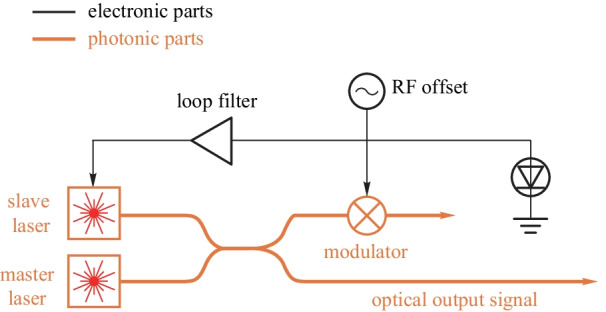
A simplified diagram of OPLL heterodyne offset locking scheme

The simplified diagram of the OPLL heterodyne offset locking scheme is shown in Fig. 17. The frequency mismatch of the slave laser and the master laser is determined by the RF offset. The closed loop feedback ensures the stable frequency relationship between the master laser and the slave laser.

The concept of OPLL is proposed in Ref. [102], and after that various OPLL schemes have been demonstrated. Compared to fiber lasers and solid-state lasers with narrow linewidths, semiconductor lasers are generally favored because of their small sizes, low costs, and high efficiencies. In Ref. [46], the first monolithically integrated optical phase-locked loop photonic integrated circuits are demonstrated. In Ref. [103], the OPLL forms the fundamental part of the analog coherent receiver scheme.

The OPLL use the electronic-photonic feedback loop to generate the high-performance optical signal at low cost. One of the main challenges is to achieve wide loop bandwidth with small loop delay to suppress the phase noise.

#### Laser frequency stabilization

The low noise stable lasers have a wide range of applications, e.g., spectroscopy, communication, metrology, and basic science [19]. The Pound–Drever–Hall laser stabilization method can fix the laser oscillation frequency. By locking the laser frequency to the frequency reference such as Mach–Zehnder interference or etalon, the phase noise of the laser is greatly reduced, and the laser is immune to environmental fluctuations.

**Fig. 18:**
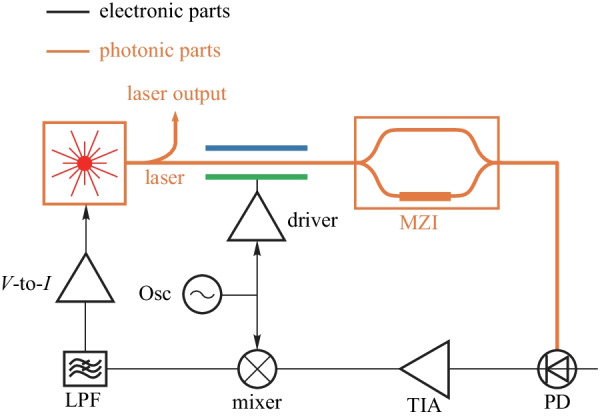
A simplified diagram of Pound–Drever–Hall laser stabilization system with MZI

**Fig. 19:**
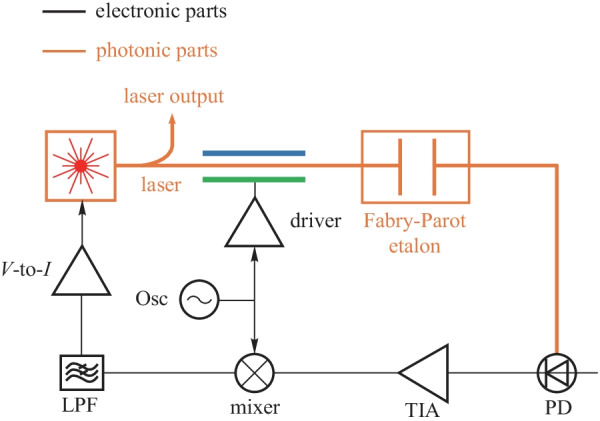
A simplified diagram of Pound–Drever–Hall laser stabilization system with etalon

The simplified diagram of the Pound–Drever–Hall laser stabilization system is shown in Figs. 18 and 19. The laser is phase-modulated with the electronic oscillator and sent to the reconfigurable MZI (or the etalon). The output optical signal of the MZI (or the etalon) is converted to an electronic signal by the photodiode. The electronic signal is further processed by the electronic system and fed back to the laser to close the PDH loop. The frequency of the laser is locked to the notch frequency of the MZI (or the etalon). The bias regulator for MZI is essential for stable oscillation.

The Pound–Drever–Hall system is proposed in Ref. [104], where the laser’s frequency is measured with a Fabry–Perot cavity, and this measurement is fed back to the laser to suppress frequency fluctuations. To make this bulky system more compact, the integrated Pound–Drever–Hall system is demonstrated in Ref. [19], where the MZI is used to measure the laser’s frequency.

The main challenge of the Pound–Drever–Hall system is to guarantee the accuracy of the frequency reference. The frequency regulator is essential to this system.

## Future perspectives

EPHIC is an emerging interdisciplinary research field. To accelerate the research progress, modeling methods and EPDA tools are required, but have not been standardized yet. Most existing works are board-level designs, and the emerging field of EPHIC is largely unexplored. For photonic signal stabilization, there have been a few designs related to wavelength stabilization. Stability problems in other dimensions (e.g., polarization and mode) are rarely studied. The multi-dimensional property of light has not fulfilled its promise at short reach and chip level, even though it has been commercialized in long-haul communications. The power-hungry DSP approach in long-haul communications is not suitable for short-reach links where energy efficiency is a key design consideration. The all-optical signal processing approach can remove the power-hungry DSP but still requires electronics in a hybrid closed-loop to control the photonic devices. It is essentially an electronics-assisted optical processing method. Increasing the bandwidth of the hybrid loop using the EPHIC is critical for further performance enhancement.

**Fig. 20:**
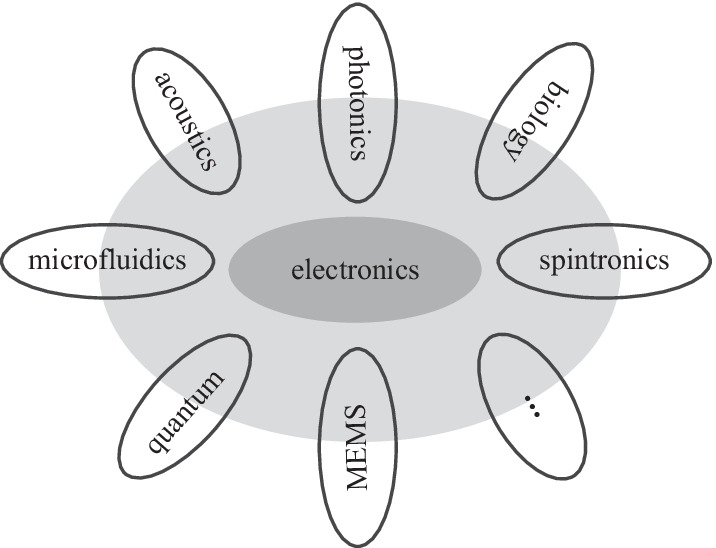
Convergence in multiple signal domains

The circuit-level convergence of electronics and photonics has just started. EPHIC with multi-dimensional signals, multiple parameters, multiple material platforms, and nonlinear photonics is worthy of further study. Currently, integrated photonics are mostly used for communications in practice. We can further extend their applications to computing and sensing. Generalizing the scalability triangle from conventional ICs to other heterogeneous ICs creates new research directions. The electronic domain is so far the only signal domain that has the complete scalability triangle. The scalability triangle does not exist in all signal domains. The existence of the scalability triangle in other signal domains (e.g., quantum and acoustic signal domain) will be an interesting problem. Other signal domains can be combined with electronics to form new types of heterogeneously-converging ICs. Currently, integrated quantum photonics is also experiencing the transition from device integration to circuit design. Similarly, integrated quantum photonics and electronics will eventually be converged. EPC forms the foundation of and is the essential step toward quantum EPC. It has been pointed out that a new abstract layer is required for quantum integrated technology [105–107]. We believe the metacircuits framework and the development experience of EPHIC will serve as a natural reference for this new abstract layer. We expect new heterogeneous regulators and new heterogeneous oscillators in these new types of heterogeneous ICs. As shown in Fig. 20, more and more signal domains will be converged.

## Conclusions

As Moore’s law approaches its end, converging is replacing scaling as the new driving force for the information industry. EPC is an important post-Moore’s law research area and is now experiencing a transition from device integration to circuit design. We have presented a metacircuits framework for constructing scalable integrated circuits, and feedback plays a central role in this framework. Combining this framework with EIC experience, we have identified and reviewed the key building blocks of EPHIC. Compact modeling and electronic-photonic design automation tools will expedite the EPC transition process. As a final remark, the experience of EIC and EPHIC can be extended to other heterogeneous circuits involving multi-physics signals.
